# In silico identification of putative druggable pockets in PRL3, a significant oncology target

**DOI:** 10.1016/j.bbrep.2024.101767

**Published:** 2024-07-01

**Authors:** Grace M. Bennett, Julia Starczewski, Mark Vincent C. dela Cerna

**Affiliations:** Department of Biochemistry, Chemistry, and Physics, Georgia Southern University, Savannah, GA, 31419, USA

## Abstract

Protein tyrosine phosphatases (PTP) have emerged as targets in diseases characterized by aberrant phosphorylations such as cancers. The activity of the phosphatase of regenerating liver 3, PRL3, has been linked to several oncogenic and metastatic pathways, particularly in breast, ovarian, colorectal, and blood cancers. Development of small molecules that directly target PRL3, however, has been challenging. This is partly due to the lack of structural information on how PRL3 interacts with its inhibitors. Here, computational methods are used to bridge this gap by evaluating the druggability of PRL3. In particular, web-based pocket prediction tools, DoGSite3 and FTMap, were used to identify binding pockets using structures of PRL3 currently available in the Protein Data Bank. Druggability assessment by molecular dynamics simulations with probes was also performed to validate these results and to predict the strength of binding in the identified pockets. While several druggable pockets were identified, those in the closed conformation show more promise given their volume and depth. These two pockets flank the active site loops and roughly correspond to pockets predicted by molecular docking in previous papers. Notably, druggability simulations predict the possibility of low nanomolar affinity inhibitors in these sites implying the potential to identify highly potent small molecule inhibitors for PRL3. Putative pockets identified here can be leveraged for high-throughput virtual screening to further accelerate the drug discovery against PRL3 and development of PRL3-directed therapeutics.

## Highlights

• The phosphatase of regenerating liver 3, PRL3, is a significant target in several cancers and in metastasis.
• There is a critical need to identify inhibitors of PRL3 with potential to be developed as antineoplastic drugs or migrastatics.
• Several putative druggable pockets are identified using a combination of computational tools which can be leveraged in virtual drug screens or structure-based drug design.
• Druggability simulations predict theoretical high-affinity binding sites.

## 1 Introduction

Aberrant cellular phosphorylation is a hallmark of several diseases including inflammation and cancers [[1], [2], [3]]. Protein phosphorylation, regulated by kinases and phosphatases, acts as a switch controlling biochemical pathways. As such, kinases and phosphatases present as significant clinical molecular targets. To date, several kinase inhibitors have been approved for various indications and kinases have been regarded as one of the most important drug targets of the 21st century [[4], [5], [6], [7], [8]]. Meanwhile, phosphatases are only recently gaining traction as therapeutic drug targets, particularly with emerging roles in cancers [[10], [11], [12], [9]]. Development of phosphatase inhibitors offer a novel approach to treatment of diseases involving dysregulated protein phosphorylation.

The phosphatase of regenerating liver (PRL), also known as the protein tyrosine phosphatase 4A (PTP4A), family is of significant interest in drug discovery as PRL expression has been correlated with cancers [[13], [14], [15], [16]]. PRL3 or PTP4A3 is the most well-studied PRL and is highly expressed in several cancer types. Its expression has been correlated with poor patient prognosis in various cancers and with increased proliferation and metastatic potential in cellular models [[17], [18], [19], [20], [21], [22], [23], [24], [25], [26]]. It has been shown to be involved in regulation of apoptosis, cellular metabolism, DNA integrity, epithelial-to-mesenchymal transition (EMT), and angiogenesis [27]. As such, PRL3 is emerging as one of the most prominent phosphatase drug targets in recent years.

Given its roles in cancers, PRL3 has been the target of several drug discovery programs leading to the identification of potential inhibitors [11,13,14,[28], [29], [30], [31], [32], [33], [34], [35]]. Pentamidine isethionate, an FDA approved anti-protozoa drug, is among the first molecules to be identified to inhibit PRLs *in vitro* [13]. High-throughput screening identified rhodanine derivatives, and one of the most potent inhibitors at the time, thienopyridone [[29], [30], [31], [32],^34^,^36^]. Elaboration of the thienopyridone scaffold led to JMS-053, currently the most potent PRL3 inhibitor [33,37,38]. Recently, JMS-053 was linked to an adamantly moiety to generate a bifunctional ER stress inducer/PRL3 inhibitor [35]. Despite the success of these campaigns, however, no small molecule inhibitor targeting PRL3 has advanced to clinical studies. The rhodanine scaffold has been considered a promiscuous binder and likely is associated with several off-target effects [37,39]. Meanwhile, the thienopyridone scaffold exhibits redox activity which potentially inhibits enzymes susceptible to oxidation, such as PRL3 [37,40]. Thus, while there a few candidate molecules, the search for inhibitors for PRL3 with potential to be developed as cancer therapeutics remains open.

To date, a few structures of PRL3 have been experimentally determined, capturing its open and closed conformations [[41], [42], [43], [44]]. The closed conformation was determined in the presence of vanadate, a general PTP inhibitor, bound to the active site [44,45]. A crystal structure of PRL3 has also been determined where the CBS-pair domain of CNNM3 is bound to the active site [41]. In this interaction, PRL3 acts as a pseudo-phosphatase, revealing a unique cellular function for the PRL3 family. While these structures have allowed for characterization of function of PRL3, a challenge remains in that none of these structures capture how PRL3 binds to small molecules, which is critical for structure-based drug design and virtual screening campaigns.

In this present work, available structural information is leveraged to identify and characterize putative druggable pockets within PRL3. The objective is to inform high throughput *in silico* screening efforts by focusing on pockets amenable for inhibitor binding. Pocket identification tools were used, along with molecular dynamics (MD) simulations using probes, to identify druggable pockets towards the development of highly specific PRL3 inhibitors.

## 2 Methods

*Protein Structures.* All analyses were performed on three structures of PRL3 taken from the Protein Data Bank: 2MBC, 1V3A, and 5TSR. These structures represent the open (1V3A) and closed (2MBC, 5TSR) conformations of PRL3 [41,44]. One of the closed conformations (5TSR) is the structure of PRL3 bound to CNNM3 domain determined by X-ray crystallography to a resolution of 3.19 Å. A closed conformation in the presence of vanadate (2MBC) was determined by solution NMR. Only the first model of the twenty in the NMR bundle was used. The open conformation (1V3A) was also determined by solution NMR and only a single conformer was deposited [42]. All these structures were used without further modification.

*Druggability simulations and analysis.* Druggability molecular dynamics simulations were set-up using the druggability suite VMD plug-in (DruGUI) and were performed using the NAMD (v. 2.14 multicore) and CHARMM22 forcefield [46,47]. PRL3 structures were placed in a box with 8 Å padding, containing explicit TIP3P water and enough chloride ions to neutralize the system. The probe composition for all runs was set at 80 % isopropanol and 20 % acetamide.

Simulations were first minimized for 2000 steps prior to a series of equilibration steps. First, the system was heated from 100 K to 300 K over 40 ps and ran at 300 K for 80 ps. Then, the system was further heated to 600 K over 60 ps, ran at 600 K for 600 ps, and cooled back to 300 K over 18 ps. During these equilibration steps, the Cα atoms were restrained by a harmonic potential with a force constant of 1 kcal/mol/A^2^. A final 600 ps unrestrained equilibration was done at 300 K. Two independent 40-ns production runs were carried out for each of the systems.

Analysis of binding hotspots was done using the built-in analysis tool in the DruGUI plug-in. The default parameters were used for the analyses of all simulations. The two simulations for each system were analyzed individually. Results were visualized using VMD, PyMol, or ChimeraX [[48], [49], [50]].

*Detection of possible binding pockets.* Two web-based tools were used to detect and identify potentially druggable pockets. The tools were chosen as they employ distinct algorithms in identifying protein pockets and are both free to use. DoGSite3 is an automated grid-based pocket detection tool included in the Protein*Plus* server (Center of Bioinformatics, University of Hamburg). It uses Difference of Gaussian (DoG) filter for pocket prediction and considers volume, hydrophobicity, enclosure, and depth [[51], [52], [53]]. Meanwhile, FTmap relies on the identification of binding hotspots of 16 small chemical probes. Docked probes go through energy-based clustering followed by consensus clustering to identify binding hotspots [54]. The prepared structures were directly uploaded onto these web tools and results were visualized with either PyMol or ChimeraX [48,49].

## 3 Results and discussion

Protein tyrosine phosphatases are emerging as significant therapeutic targets, particularly in cancers [3,55]. Among the PRL family, PRL3 is the most well-studied and most targeted by on-going drug discovery efforts. While several inhibitors have been identified, there is currently no information on how PRL3 interacts with any small molecule. That is, there is currently no known structure of PRL3 in complex with any inhibitor. Information on the interaction of a protein target and inhibitors is critical for the rational improvement of these inhibitors and the design of new ones [56]. In the absence of these structures, any information on putative binding pockets is crucial to *in silico* drug screening efforts [51,54,56,57]. This study aims to evaluate the druggability of PRL3 and to identify and characterize putative druggable pockets which will further inform drug discovery and development against this important target. To maximize the likelihood of identifying a druggable pocket, multiple structures (open, closed, and pseudo-substrate-bound, Supplementary Fig. 1) are used along with multiple computational tools. Two web-based binding pocket detection tools with unique detection algorithms were used, along with molecular dynamics druggability simulations.

**Fig. 1:**
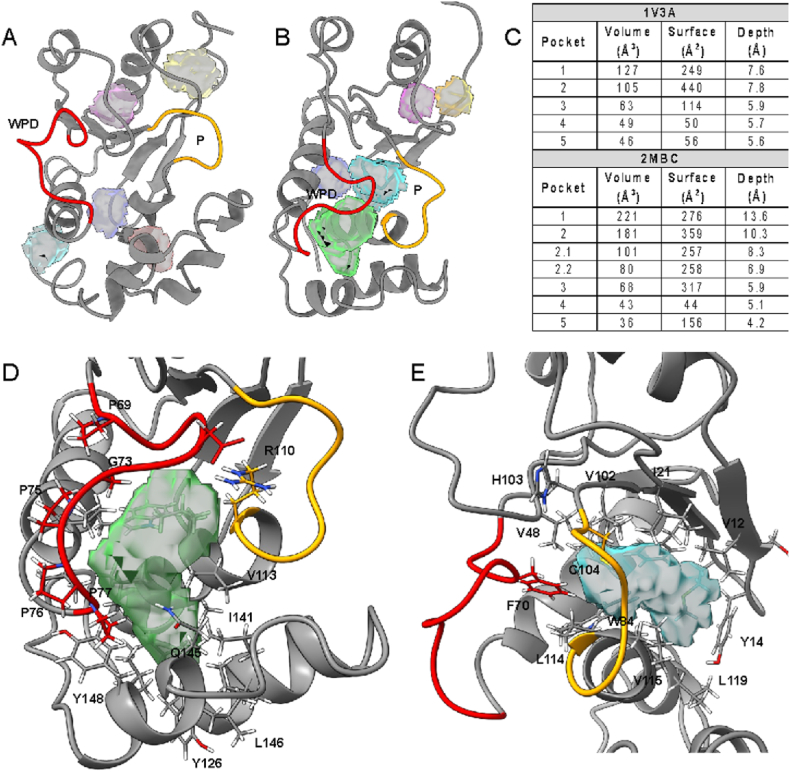
**Pockets identified by DoGSite3.** Several small pockets were identified in the open (1V3A, A, C) and closed conformations (2MBC, B, C) of PRL3. Pocket parameters, including volume, surface, and depth are summarized (C, Supplementary Table 1). Two major pockets are identified in the closed conformation (Pockets 1 and 2 in 2MBC) cradled near the active site loops (red and orange). (For interpretation of the references to color in this figure legend, the reader is referred to the Web version of this article.)

DoGSite3 is an improvement on DoGSiteScorer that better handles binding site boundary using a depth-first search [51]. It identified several putative pockets in all three conformations analyzed (Supplementary Tables 1 and 2, Fig. 1). Five putative binding pockets were identified in the open conformation ranging from 46 to 127 Å^3^ in volume (Fig. 1A–C). The 127 Å^3^ pocket has a depth of 7.6 Å and is the largest in this conformation. The vanadate-bound, closed conformation has larger pockets with two prominent ones having volumes of 221 Å^3^ and 181 Å^3^ (Fig. 1B and C). These are adjacent to the active site loops, the WPD and P loops, respectively. These pockets are also the deepest ones detected at 13.6 Å for the WPD-adjacent and 10.3 Å and P-adjacent pockets (Fig. 1D and E). These pockets involve the active site residues D72, C104, and R110 and are highly hydrophobic (64 % and 84 % hydrophobic residues, respectively, Fig. 1D and E, Supplementary Table 1). The pseudo-substrate-bound structure did not show comparable binding pockets (Supplementary Table 1). Interestingly, using the older version of DoGSite3, two adjacent pockets that cover the entire active site are identified, in addition to several others, with a combined volume of 1030 Å^3^ (Supplementary Table 2). This is not surprising considering that PRL3 has a shallow active site and DoGSite3 factors in the depth of the pocket [42,43].

**Fig. 2:**
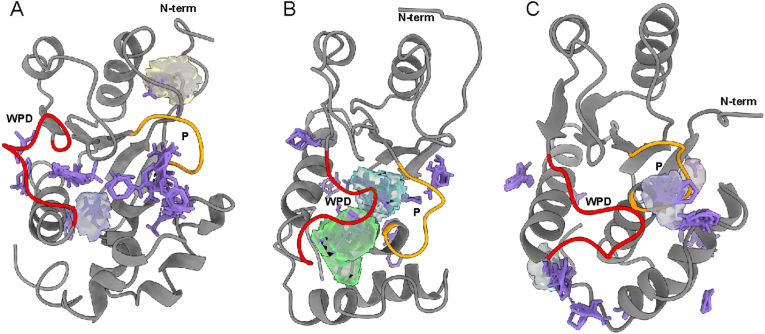
**Fragment hotspots determined by FTmap.** Several hotspots for the various probes (all colored purple to emphasize positions regardless of probe identity) were identified in the open (1V3A, A), vanadate-bound (2MBC, B), and pseudo-substrate bound (5TSR, C) conformations. Where there is overlap, the DoGSite3-predicted pockets are also shown as surfaces. (For interpretation of the references to color in this figure legend, the reader is referred to the Web version of this article.)

**Fig. 3:**
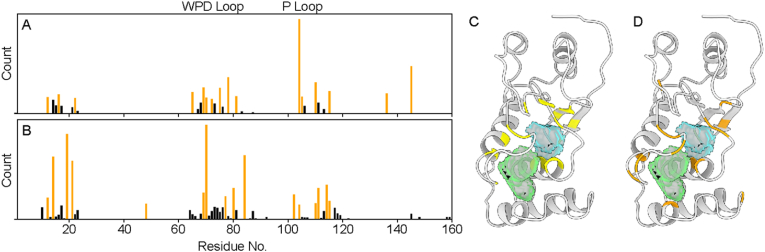
**Residue level interaction data from FTmap.** Raw counts of hydrogen bonding (A) and non-bonded (B) interactions in the closed conformation (2MBC). Active site loops are highlighted (WPD, yellow and P, orange) along with significant interaction counts, defined as 15 % of the highest counts or more (orange bars). These top hydrogen bonding (C) and non-bonding (D) interactions are mapped onto the structure along with the primary pockets identified in DoGSite3 (yellow and blue surfaces). (For interpretation of the references to color in this figure legend, the reader is referred to the Web version of this article.)

Another approach to identifying putative binding pockets in a protein is the computational analogue to fragment screening [58,59]. One such implementation is FTmap, which performs rigid docking of 16 small molecule probes to identify clusters or hotspots whicåh correspond to possible drug binding pockets [54]. Using FTmap, several potential binding pockets were identified in all three conformations studied, some of which overlap with pockets identified by DoGSite3 (Fig. 2). While the active site in the open conformation does not meet the depth criteria of DoGSite3, pockets identified by DoGSiteScorer within the active site overlap with FTmap hotspots (Supplementary Fig. 2). Fragment binding predicted by FTmap may imply that, while shallow, it is still possible to design molecules that bind to this pocket. Interestingly, a small pocket near the N-terminus also overlaps with an FTmap hotspot (Fig. 2A). Meanwhile, for the vanadate-bound conformation, the biggest FTmap clusters of probes overlap with the biggest pockets identified by DoGSite3, adjacent to the WPD and P loops (Fig. 2B). Similarly, several hotspots were identified for the pseudo-substrate-bound conformation, including some that overlap with the smaller DoGSite3 pockets (Fig. 2C). In addition to identifying hotspots from consensus clusters based on docked probes, FTmap also quantifies the non-bonded and hydrogen bonding interaction between residues and the probes (Fig. 3, Supplementary Fig. 3). For the vanadate-bound closed conformation, there is significant involvement from the active site loops (WPD and P) in binding the hotspots (Fig. 3A and B). The major interacting residues (15 % of the top residue count or higher) map to residues that are identified in DoGSite3 as well (Fig. 3C and D). Moreover, there is significant non-bonded interactions identified near the N-terminus (residues 10–20, Fig. 3B) which is potentially another pocket, albeit significantly smaller. This area was also identified by DogSite3 (Fig. 1B). Active site involvement is not as prominent in the open conformation, although there is potential for significant interactions adjacent to the P loop (residues 110–120, Supplementary Figs. A and B). This is again reflective of the shallow binding pocket of PRL3; though interestingly, several hotspots are detected in that shallow pocket (Fig. 2A).

Overall, these two approaches identified pockets of potential relevance. It is noteworthy that based on these two techniques, the vanadate-bound closed conformation reveals the most promising binding pockets (Fig. 1, Fig. 2B, Supplementary Table 1). These pockets have sufficient volume and depth to be identified by DoGSite3, and is supported by FTmap's clusters and interaction data. There are also some sites in the open conformation identified by both methods, although they are significantly smaller (Fig. 2A). The difference in pocket identification algorithm also allows FTmap to identify potential hotspots within the active site of the open conformation that is missed by DoGSite3 (Fig. 2A, Supplemental Fig. 2). While these pockets are capable of hydrogen bonding, majority of the residues are hydrophobic (Supplementary Table 2).

**Fig. 4:**
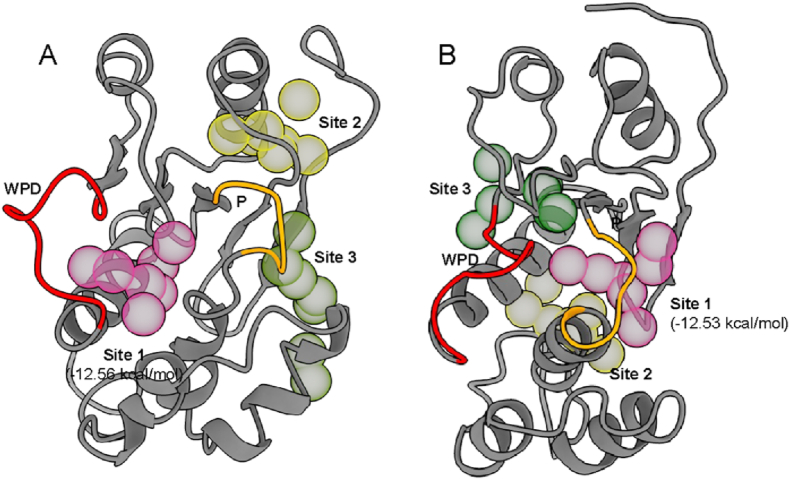
**Druggability simulations identify probe hotspots.** Top 3 hotspots from DruGUI druggability simulations for the open (1V3A, A) and vanadate-bound closed (2MBC, B) conformation of PRL3.

To further analyze these pockets, a molecular dynamics druggability simulation was performed using the DruGUI VMD plugin [47,50]. This offers yet another unique approach to validate the identified pockets. Like FTmap, druggability simulations makes use of small molecule probes to identify potential druggable pockets [54]. An MD simulation in explicit water in the presence of molecular probes is used to identify hotspots and estimate maximal predicted binding affinity at a site, as opposed to the rigid docking method [47]. Several hotspots were identified by the druggability simulations (Supplementary Table 3), particularly in the open and vanadate-bound closed conformation. The top hotspots, based on predicted lowest binding energy (or highest binding affinity) for the open and vanadate-bound conformations capture some of the previously identified pockets (Table 1). Several hotspots have highly desirable predicted maximum binding affinities from low nanomolar to <100 μM, indicating that these might be promising druggable pockets. The top predicted binding pocket in the open conformation, for instance, is the shallow active site pocket (Fig. 4A) with a theoretical strongest binding affinity at the nanomolar range. A similarly potentially high affinity binding pocket is detected in the closed conformation, adjacent to the P loop (Fig. 4B). This site roughly corresponds with the P loop-adjacent site identified by DoGSite3 and FTmap (Fig. 2, Fig. 3). These druggability simulations further support the druggability of the major pockets identified in both conformations.

**Table 1:** Hotspots predicted by druggability simulations.

1V3A			
Site	Binding Energy (kcal/mol)	Affinity (nM)	Volume (Å^3^)
1	−12.56	0.7	436
2	−11.00	9.5	401
3	−9.19	200	380

2MBC			
Site	Binding Energy (kcal/mol)	Affinity (nM)	Volume (Å^3^)
1	−12.53	0.7	398
2	−11.59	3.6	438
3	−11.34	5.4	403

Overall, this study has identified druggable binding pockets, including the active site and most notably, a binding pocket in the closed conformation that is adjacent to the active site P loop. As phosphatases have highly conserved active site loops, the pocket identified in the closed conformation (Site 1 in Fig. 4B) presents a promising druggable pocket for development of non-competitive inhibitors. Residues that line these pockets have also been identified for use in high-throughput flexible side chain docking simulations [[62], [63], [64]]. Druggability simulations predict high affinity binders in several pockets. While this is a theoretical maximum, this provides hope for future drug discovery programs to identify potent PRL3 inhibitors. The pockets identified here also support and validate previous predictions. For instance, a previous virtual screening attempt identified inhibitors that bind the shallow active site [32]. The thienopyridone scaffold was also docked in the closed conformation near the WPD loop, near one of the sites identified in the present study [38]. Blind docking of FDA-approved drugs similarly identified roughly the same pockets in both the open and closed conformations [60]. In the absence of experimental structures of PRL3 in complex with inhibitors, these computational studies provide invaluable information that will guide drug design efforts against this important therapeutic target.

## 4 Conclusion

PRL3 is a protein tyrosine phosphatase (PTP) that has emerged as a significant oncology drug target. While PTPs have historically been tagged ‘undruggable,’ this family is slowly shedding this identity [[9], [12],^61^]. This study contributes to further advancing PRL3-targeted drug discovery by identifying and analyzing potential druggable pockets. Three unique computational tools identified consensus sites, as well as unique sites, that could be the focus of virtual drug screening. Knowledge on the residues that might be involved in drug binding can be used in conjunction with docking with flexible sidechains [[62], [63], [64]]. Additionally, binding of candidate molecules to these pockets can be further analyzed using advanced computational simulations which have been shown to provide more accurate binding energies for biological systems. Methods such as the density functional-based tight binding (DFTB), for instance, have been used to evaluate the binding of candidate molecules from virtual screening campaigns against the SARS-CoV-2 and HIV proteases [65,66]. Similar studies can be performed on PRL3 towards characterizing its interaction with potential inhibitors. Furthermore, this study contributes to further supporting the druggability of PRL3 – that perhaps more high affinity inhibitors are just soon to be discovered.

## Funding

Mark dela Cerna reports financial support was provided by the Vertically Integrated Projects (VIP) Program at Georgia Southern University. Grace Bennett reports financial support was provided by American Society for Biochemistry and Molecular Biology. If there are other authors, they declare that they have no known competing financial interests or personal relationships that could have appeared to influence the work reported in this paper.

## CRediT authorship contribution statement

**Grace M. Bennett:** Writing – review & editing, Writing – original draft, Visualization, Investigation, Formal analysis, Data curation, Conceptualization. **Julia Starczewski:** Writing – original draft, Visualization, Data curation. **Mark Vincent C. dela Cerna:** Writing – review & editing, Visualization, Validation, Project administration, Funding acquisition, Formal analysis, Conceptualization.

## Declaration of competing interest

The authors declare the following financial interests/personal relationships which may be considered as potential competing interests:

Grace Bennett reports financial support was provided by 10.13039/100013616American Society for Biochemistry and Molecular Biology. If there are other authors, they declare that they have no known competing financial interests or personal relationships that could have appeared to influence the work reported in this paper.

## Data Availability

Data will be made available on request.

## References

[1] Cohen P. (2001). The role of protein phosphorylation in human health and disease. Eur. J. Biochem..

[2] Ardito F., Giuliani M., Perrone D., Troiano G., Muzio L.L. (2017). The crucial role of protein phosphorylation in cell signaling and its use as targeted therapy. Int. J. Mol. Med..

[3] Östman A., Hellberg C., Böhmer F.D. (2006). Protein-tyrosine phosphatases and cancer. Nat. Rev. Cancer.

[4] Roskoski R. (2015). A historical overview of protein kinases and their targeted small molecule inhibitors. Pharmacol. Res..

[5] Overington J.P., Al-Lazikani B., Hopkins A.L. (2006). How many drug targets are there?. Nat. Rev. Drug Discov..

[6] Cohen P., Cross D., Jänne P.A. (2021). Kinase drug discovery 20 years after imatinib: progress and future directions. Nat. Rev. Drug Discov..

[7] Cohen P., Alessi D.R. (2013). Kinase drug discovery – what's next in the field?. ACS Chem. Biol..

[8] Cohen P. (2002). Protein kinases — the major drug targets of the twenty-first century?. Nat. Rev. Drug Discov..

[9] Dang C.V., Reddy E.P., Shokat K.M., Soucek L. (2017). Drugging the “undruggable” cancer targets. Nat. Rev. Cancer.

[10] Zhang Z.-Y. (2016). Drugging the undruggable: therapeutic potential of targeting protein tyrosine phosphatases. Accounts Chem. Res..

[11] Lazo J.S., McQueeney K.E., Sharlow E.R. (2017). New approaches to difficult drug targets: the phosphatase story. Slas Discov..

[12] Stanford S.M., Bottini N. (2017). Targeting tyrosine phosphatases: time to end the stigma. Trends Pharmacol. Sci..

[13] Pathak M.K., Dhawan D., Lindner D.J., Borden E.C., Farver C., Yi T. (2002). Pentamidine is an inhibitor of PRL phosphatases with anticancer activity. Mol. Cancer Therapeut..

[14] Wei M., Korotkov K.V., Blackburn J.S. (2018). Targeting phosphatases of regenerating liver (PRLs) in cancer. Pharmacol. Therapeut..

[15] Bessette D.C., Qiu D., Pallen C.J. (2008). PRL PTPs: mediators and markers of cancer progression. Cancer Metastasis Rev..

[16] Hardy S., Kostantin E., Hatzihristidis T., Zolotarov Y., Uetani N., Tremblay M.L. (2018). Physiological and oncogenic roles of the PRL phosphatases. FEBS J..

[17] Beekman R., Valkhof M., Erkeland S.J., Taskesen E., Rockova V., Peeters J.K., Valk P.J.M., Löwenberg B., Touw I.P. (2011). Retroviral integration mutagenesis in mice and comparative analysis in human AML identify reduced PTP4A3 expression as a prognostic indicator. PLoS One.

[18] Dai N., Lu A.-P., Shou C.-C., Li J.-Y. (2009). Expression of phosphatase regenerating liver 3 is an independent prognostic indicator for gastric cancer. World J. Gastroenterol..

[19] Mayinuer A., Yasen M., Mogushi K., Obulhasim G., Xieraili M., Aihara A., Tanaka S., Mizushima H., Tanaka H., Arii S. (2013). Upregulation of protein tyrosine phosphatase type IVA member 3 (PTP4A3/PRL-3) is associated with tumor differentiation and a poor prognosis in human hepatocellular carcinoma. Ann. Surg Oncol..

[20] Qu S., Liu B., Guo X., Shi H., Zhou M., Li L., Yang S., Tong X., Wang H. (2014). Independent oncogenic and therapeutic significance of phosphatase PRL-3 in FLT3-ITD–negative acute myeloid leukemia. Cancer.

[21] Radke I., Götte M., Kersting C., Mattsson B., Kiesel L., Wülfing P. (2006). Expression and prognostic impact of the protein tyrosine phosphatases PRL-1, PRL-2, and PRL-3 in breast cancer. Br. J. Cancer.

[22] Ren T., Jiang B., Xing X., Dong B., Peng L., Meng L., Xu H., Shou C. (2009). Prognostic significance of phosphatase of regenerating liver-3 expression in ovarian cancer. Pathol. Oncol. Res..

[23] Saha S., Bardelli A., Buckhaults P., Velculescu V.E., Rago C., Croix B. St, Romans K.E., Choti M.A., Lengauer C., Kinzler K.W., Vogelstein B. (2001). A phosphatase associated with metastasis of colorectal cancer. Science.

[24] Wu X., Zeng H., Zhang X., Zhao Y., Sh H., Ge X., Zhang M., Gao X., Xu Q. (2010). Phosphatase of regenerating liver-3 promotes motility and metastasis of mouse melanoma cells. Am. J. Pathol..

[25] Kato H., Semba S., Miskad U.A., Seo Y., Kasuga M., Yokozaki H. (2004). High expression of PRL-3 promotes cancer cell motility and liver metastasis in human colorectal cancer: a predictive molecular marker of metachronous liver and lung metastases. Clin. Cancer Res..

[26] Polato F., Codegoni A., Fruscio R., Perego P., Mangioni C., Saha S., Bardelli A., Broggini M. (2005). PRL-3 phosphatase is implicated in ovarian cancer growth. Clin. Cancer Res..

[27] Chia P.L., Ang K.H., Thura M., Zeng Q. (2023). PRL3 as a therapeutic target for novel cancer immunotherapy in multiple cancer types. Theranostics.

[28] Jiang Z.-X., Zhang Z.-Y. (2008). Targeting PTPs with small molecule inhibitors in cancer treatment. Cancer Metastasis Rev..

[29] Vintonyak V.V., Waldmann H., Rauh D. (2011). Using small molecules to target protein phosphatases. Bioorg. Med. Chem..

[30] Ahn J.H., Kim S.J., Park W.S., Cho S.Y., Ha J.D., Kim S.S., Kang S.K., Jeong D.G., Jung S.-K., Lee S.-H., Kim H.M., Park S.K., Lee K.H., Lee C.W., Ryu S.E., Choi J.-K. (2006). Synthesis and biological evaluation of rhodanine derivatives as PRL-3 inhibitors. Bioorg. Med. Chem. Lett..

[31] Min G., Lee S.-K., Kim H.-N., Han Y.-M., Lee R.-H., Jeong D.G., Han D.C., Kwon B.-M. (2013). Rhodanine-based PRL-3 inhibitors blocked the migration and invasion of metastatic cancer cells. Bioorg. Med. Chem. Lett.

[32] Hoeger B., Diether M., Ballester P.J., Köhn M. (2014). Biochemical evaluation of virtual screening methods reveals a cell-active inhibitor of the cancer-promoting phosphatases of regenerating liver. Eur. J. Med. Chem..

[33] Tasker N.R., Rastelli E.J., Burnett J.C., Sharlow E.R., Lazo J.S., Wipf P. (2019). Tapping the therapeutic potential of protein tyrosine phosphatase 4A with small molecule inhibitors. Bioorg. Med. Chem. Lett..

[34] Lin L., Lu L., Yuan C., Wang A., Zhu M., Fu X., Xing S. (2021). The dual inhibition against the activity and expression of tyrosine phosphatase PRL-3 from a rhodanine derivative. Bioorg. Med. Chem. Lett..

[35] Rastelli E.J., Sannino S., Hart D.J., Sharlow E.R., Lazo J.S., Brodsky J.L., Wipf P. (2021). Synthesis and evaluation of bifunctional PTP4A3 phosphatase inhibitors activating the ER stress pathway. Bioorg. Med. Chem. Lett..

[36] Daouti S., Li W., Qian H., Huang K.-S., Holmgren J., Levin W., Reik L., McGady D.L., Gillespie P., Perrotta A., Bian H., Reidhaar-Olson J.F., Bliss S.A., Olivier A.R., Sergi J.A., Fry D., Danho W., Ritland S., Fotouhi N., Heimbrook D., Niu H. (2008). A selective phosphatase of regenerating liver phosphatase inhibitor suppresses tumor cell anchorage-independent growth by a novel mechanism involving p130Cas cleavage. Cancer Res..

[37] Salamoun J.M., McQueeney K.E., Patil K., Geib S.J., Sharlow E.R., Lazo J.S., Wipf P. (2016). Photooxygenation of an amino-thienopyridone yields a more potent PTP4A3 inhibitor. Org. Biomol. Chem..

[38] McQueeney K.E., Salamoun J.M., Burnett J.C., Barabutis N., Pekic P., Lewandowski S.L., Llaneza D.C., Cornelison R., Bai Y., Zhang Z.-Y., Catravas J.D., Landen C.N., Wipf P., Lazo J.S., Sharlow E.R. (2017). Targeting ovarian cancer and endothelium with an allosteric PTP4A3 phosphatase inhibitor. Oncotarget.

[39] Mendgen T., Steuer C., Klein C.D. (2012). Privileged scaffolds or promiscuous binders: a comparative study on rhodanines and related heterocycles in medicinal chemistry. J. Med. Chem..

[40] Zhang Z., Kozlov G., Chen Y.S., Gehring K. (2019). Mechanism of thienopyridone and iminothienopyridinedione inhibition of protein phosphatases. Medchemcomm.

[41] Zhang H., Kozlov G., Li X., Wu H., Gulerez I., Gehring K. (2017). PRL3 phosphatase active site is required for binding the putative magnesium transporter CNNM3. Sci. Rep.-uk.

[42] Kim K.-A., Song J.-S., Jee J., Sheen M.R., Lee C., Lee T.G., Ro S., Cho J.M., Lee W., Yamazaki T., Jeon Y.H., Cheong C. (2004). Structure of human PRL‐3, the phosphatase associated with cancer metastasis. FEBS Lett..

[43] Kozlov G., Cheng J., Ziomek E., Banville D., Gehring K., Ekiel I. (2004). Structural insights into molecular function of the metastasis-associated phosphatase PRL-3. J. Biol. Chem..

[44] Jeong K.-W., Kang D.-I., Lee E., Shin A., Jin B., Park Y.-G., Lee C.-K., Kim E.-H., Jeon Y.H., Kim E.E., Kim Y. (2014). Structure and backbone dynamics of vanadate-bound PRL-3: comparison of 15N nuclear magnetic resonance relaxation profiles of free and vanadate-bound PRL-3. Biochemistry.

[45] Swarup G., Cohen S., Garbers D.L. (1982). Inhibition of membrane phosphotyrosyl-protein phosphatase activity by vanadate. Biochem. Biophys. Res. Commun..

[46] Phillips J.C., Hardy D.J., Maia J.D.C., Stone J.E., Ribeiro J.V., Bernardi R.C., Buch R., Fiorin G., Hénin J., Jiang W., McGreevy R., Melo M.C.R., Radak B.K., Skeel R.D., Singharoy A., Wang Y., Roux B., Aksimentiev A., Luthey-Schulten Z., Kalé L.V., Schulten K., Chipot C., Tajkhorshid E. (2020). Scalable molecular dynamics on CPU and GPU architectures with NAMD. J. Chem. Phys..

[47] Bakan A., Nevins N., Lakdawala A.S., Bahar I. (2012). Druggability assessment of allosteric proteins by dynamics simulations in the presence of probe molecules. J. Chem. Theor. Comput..

[48] L. Schrödinger, The PyMOL Molecular Graphics System, Versioñ1.8.

[49] Pettersen E.F., Goddard T.D., Huang C.C., Meng E.C., Couch G.S., Croll T.I., Morris J.H., Ferrin T.E. (2021). UCSF ChimeraX: structure visualization for researchers, educators, and developers. Protein Sci..

[50] Humphrey W., Dalke A., Schulten K. (1996). VMD: visual molecular dynamics. J. Mol. Graph..

[51] Graef J., Ehrt C., Rarey M. (2023). Binding site detection remastered: enabling fast, robust, and reliable binding site detection and descriptor calculation with DoGSite3. J. Chem. Inf. Model..

[52] Volkamer A., Griewel A., Grombacher T., Rarey M. (2010). Analyzing the topology of active sites: on the prediction of pockets and subpockets. J. Chem. Inf. Model..

[53] Volkamer A., Kuhn D., Grombacher T., Rippmann F., Rarey M. (2012). Combining global and local measures for structure-based druggability predictions. J. Chem. Inf. Model..

[54] Kozakov D., Grove L.E., Hall D.R., Bohnuud T., Mottarella S.E., Luo L., Xia B., Beglov D., Vajda S. (2015). The FTMap family of web servers for determining and characterizing ligand-binding hot spots of proteins. Nat. Protoc..

[55] Zhang Z.-Y. (2001). Protein tyrosine phosphatases: prospects for therapeutics. Curr. Opin. Chem. Biol..

[56] Leis S., Schneider S., Zacharias M. (2010). In silico prediction of binding sites on proteins. Curr. Med. Chem..

[57] Liao J., Wang Q., Wu F., Huang Z. (2022). In silico methods for identification of potential active sites of therapeutic targets. Molecules.

[58] Klages J., Coles M., Kessler H. (2006). NMR-based screening: a powerful tool in fragment-based drug discovery. Analyst.

[59] Murray C.W., Rees D.C. (2009). The rise of fragment-based drug discovery. Nat. Chem..

[60] Rivas D.R., Cerna M.V.C.D., Smith C.N., Sampathi S., Patty B.G., Lee D., Blackburn J.S. (2021). A screen of FDA-approved drugs identifies inhibitors of protein tyrosine phosphatase 4A3 (PTP4A3 or PRL-3). Sci. Rep..

[61] Lazo J.S., Sharlow E.R. (2015). Drugging undruggable molecular cancer targets. Annu. Rev. Pharmacol..

[62] Jaghoori M.M., Bleijlevens B., Olabarriaga S.D. (2016). 1001 ways to run AutoDock Vina for virtual screening. J. Comput. Aided Mol. Des..

[63] Meiler J., Baker D. (2006). ROSETTALIGAND: protein–small molecule docking with full side‐chain flexibility. Proteins: Struct., Funct., Bioinf..

[64] Shin W.-H., Seok C. (2012). GalaxyDock: protein–ligand docking with flexible protein side-chains. J. Chem. Inf. Model..

[65] Allec S.I., Sun Y., Sun J., Chang C.-E.A., Wong B.M. (2019). Heterogeneous CPU+GPU-enabled simulations for DFTB molecular dynamics of large chemical and biological systems. J. Chem. Theor. Comput..

[66] Sepay N., Chakrabarti S., Afzal M., Alarifi A., Maj D. (2022). Identification of 4-acrylamido-*N*-(pyridazin-3-yl)benzamide as anti-COVID-19 compound: a DFTB molecular docking, and molecular dynamics study. RSC Adv..

